# Gene-targeted embryonic stem cells: real-time PCR assay for estimation of the number of neomycin selection cassettes

**DOI:** 10.1186/1480-9222-13-10

**Published:** 2011-10-28

**Authors:** Cecilia Mancini, Erika Messana, Emilia Turco, Alessandro Brussino, Alfredo Brusco

**Affiliations:** 1 Department of Genetics, Biology and Biochemistry, University of Torino, Torino, Italy; 2 S.C.D.U. Medical Genetics, A.O.U. San Giovanni Battista, Torino, Italy; 3 Molecular Biotechnology Center, Torino, Italy

## Abstract

In the preparation of transgenic murine ES cells it is important to verify the construct has a single insertion, because an ectopic neomycin phosphortransferase positive selection cassette (*NEO*) may cause a position effect. During a recent work, where a knockin SCA28 mouse was prepared, we developed two assays based on Real-Time PCR using both SYBR Green and specific minor groove binder (MGB) probes to evaluate the copies of *NEO *using the comparative delta-delta Ct method versus the *Rpp30 *reference gene.

We compared the results from Southern blot, routinely used to quantify *NEO *copies, with the two Real-Time PCR assays. Twenty-two clones containing the single *NEO *copy showed values of 0.98 ± 0.24 (mean ± 2 S.D.), and were clearly distinguishable from clones with two or more *NEO *copies.

This method was found to be useful, easy, sensitive and fast and could substitute for the widely used, but laborious Southern blot method.

## 

Two methods are available for the introduction and modification of mouse genomic DNA sequences: (i) microinjection of one or more transgenes into the pronucleus of a fertilized mouse oocyte, which usually leads to random incorporation into the genome as head-to-tail concatamers of 1-1000 units, or (ii) the use of constructs that undergo a site-specific recombination in embryonic stem cells (ES) in order to disrupt the function of a target gene (knockout) or to mutate a gene (knockin). Modified ES cells are then injected into the blastocyst [1]. In the latter case, the production of knockout or knockin ES cells is obtained through gene targeting by homologous recombination. In this work, ES cells were transfected by electroporation with a construct containing a specific genomic sequence harbouring the required mutation, along with the neomycin phophortransferase positive selection cassette (NEO) for selection of positive recombinants, flanked by two homology sequences ("arms") driving the recombination [2,3]. Homologous recombination occurs in a small number of transfected cells, resulting in the introduction of the mutation present in the targeting construct into the gene of interest. However, despite the presence of the two "arms", there may be a variable number of random integrations that may cause a position effect [4-6]. To identify the mutant ES cell clones to be microinjected, two Southern blots are usually performed: one to detect ES clones in which homologous recombination has occurred, and the other to verify the number of NEO cassettes. Usually between two and three hundred clones are analysed: useful clones are routinely just 1 - 2% of the total. This low percentage is mainly due to the event of the vector being inserted in ectopic sites.

One member of our group is responsible for a facility within the Molecular Biotechnology Center in Torino, aimed at the preparation of transgenic mice using recombinant ES cells. In routine work, it became necessary to have a rapid test to exclude the presence of additional copies of the *NEO *cassette in ES clones in which homologous recombination was successfully obtained. Here we describe a screening method using a rapid semi-quantitative real-time PCR, which was validated on ES clones with different *NEO *copies (0, 1, 2, > 2 copies), previously assessed by Southern blot.

From one of the projects involving the preparation of recombinant mice, we selected 45 genomic DNA extracted from ES clones that then underwent Southern blot screening. DNA extraction was performed using standard phenol-chloroform method [7]. Southern blot was performed using standard conditions for gel run, transfer and hybridisation. A *NEO *probe of 773 bp was used to evaluate the number of transgenic plasmid insertions.

As an alternative or complementary method to assess the *NEO *cassette copy number, we set up an assay based on quantitative real-time PCR (qPCR). The assay was performed with two protocols on an ABI 7500 Fast instrument (Applied Biosystems, Foster City, CA, USA); data were analyzed using the 7500 software. The two approaches were: (i) an MGB-based assay (MGB-assay), and (ii) a SYBR Green-based assay (SYBR-assay) (Applied Biosystems). Using the Primer Express software (Applied Biosystems) we designed specific PCR primers to amplify 62 bp of highly conserved sequences between two different *NEO *cassette-containing plasmids PL451 and PL452 (http://web.ncifcrf.gov/research/brb/recombineeringInformation.aspx). The mouse *Rpp30 *gene (63 bp, RefSeq NM_019428.3) was used as a reference gene. This gene is orthologous to the human *RNaseP*, widely used as a copy number reference in qPCR assay. Real-time PCR conditions and primer sequences are reported in Figure 1.

**Figure 1:**
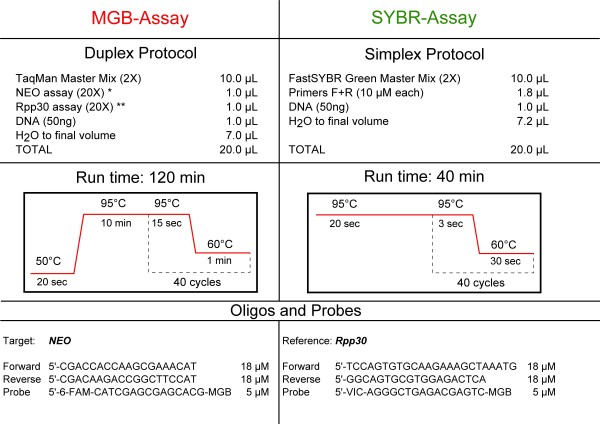
**Real-time PCR protocols for MGB- (left) and SYBR-assay (right)**. Protocols and run conditions that were used for MGB- and SYBR- assays are described in the upper part. Below are the oligo sequences and oligo concentrations used to prepare the *NEO *and *Rpp30 *assays. For the MGB-assay we prepared two (20×) mixes, one (*) for the target gene (*NEO) *and one (**) for the reference gene (*Rpp30*). For both assays, the final concentration in each reaction was 900 nM for oligos, 250 nM for probes (MGB-assay only). The MGB assay was prepared using TaqMan 2× Master Mix (Applied Biosystems): a standard run protocol of two hours was used. The SYBR-assay was performed with Fast SYBR Green 2× Master Mix (Applied Biosystems); the fast protocol reported took only 40 min.

In the MGB-assay we combined the two *NEO *and *Rpp30 *assays in a duplex PCR including two internal Taqman-MGB probes (5'-FAM labelled for *NEO *and 5'-VIC labelled for *Rpp30*) (Figure 1). Each sample was consistently run in triplicate with a blank well to check for contaminations.

In the SYBR-assay the two reactions were run in separate wells, using a Fast SYBR green mix (Applied Biosystems). The efficiency of each assay was verified with a standard curve starting from 100 ng of mouse DNA, using four serial dilutions from 1:1 to 1:8. The MGB-assay had 80% efficiency, whereas SYBR-assay gave 93% efficiency.

Gene copy-number was calculated using the comparative delta-delta Ct method [8]. In each experiment, we normalized the ΔCt of the sample to the mean ΔCt of three ES clones with a single *NEO *copy verified using Southern blot; these values (named *nNEO*) were expected to be ~ "1" in the case of a single copy of *NEO*, ~ "2" in the case of two copies of *NEO*, and "n" for "n" copies of *NEO*.

Using Southern blot, twenty-two of the 45 ES clones showed one copy of *NEO*, and 23 clones had multiple *NEO *copies. We analyzed all samples using both of the two real-time PCR assays (Figure 2). The 22 clones containing a single *NEO *copy showed a *nNEO *value of 0.98 ± 0.24 (mean ± 2 S.D.) (range 0.81-1.20) for the MGB-assay and 1.01 ± 0.24 (range 0.80-1.24) for the SYBR-assay (Figure 2A). As expected for a single copy of *NEO*, the value of *nNEO *was between 0.8 and 1.2. In a control DNA with two *NEO *copies, the value of *nNEO *was 2.2 for the MGB-assay and 2.4 for the SYBR-assay (Figure 2A).

**Figure 2:**
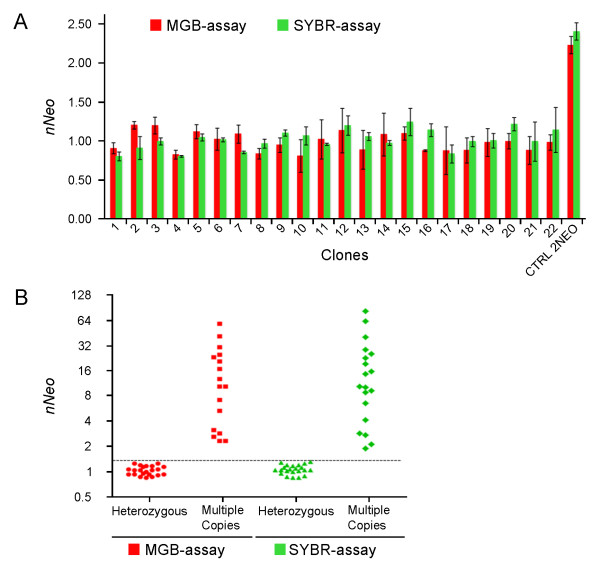
**Quantitative real-time PCR validation and results for *NEO *copy number determination, using MGB-assay (in red) and SYBR-assay (in green)**. (A) Twenty-two ES clones (abscissa, numbers from 1 to 22) showed a *nNEO *value between 0.8 and 1.2 (ordinate). One genomic DNA from a homozygous knockout mouse was used as control (CTRL 2*NEO*, *nNEO *= 2.2). Error bars indicate triplicates delta-delta Ct standard deviations. (B) Real-time PCR results: 45 ES clones are plotted on the graph, for each assay using a log_2 _scale. The dotted line indicates a *nNEO *threshold of 1.2, the upper cutoff for considering the presence of a single *NEO *copy. Samples tested with either of the two assays had similar *nNEO *values, as shown by the similar population plots.

The remaining 23 ES clones (≥ 2 *NEO *copies) showed a variable number of *nNEO *from ~2 to > 60 (Figure 2B). Comparing results of the two qPCR assays for the same sample, we saw that the values of *nNEO *had a minimal variability. Although the *nNEO *number probably reflects the amount of cassettes, calculating their exact number was beyond our scope. Curiously, we found high copy-number insertions that reached up to 60; this could be explained by the presence of concatamers of the vector, rather than multiple insertions in the mouse genome.

Southern blot analysis has long been the reference method for the detection of *NEO *copy number in ES clones. However, this method requires large amounts of DNA samples, as well as being laborious, time-consuming, and sometimes difficult to interpret. Our experience in transgenic mouse model preparation has led to the conclusion that having an alternative method is highly favourable. We propose a semi-quantitative real-time PCR method, that only requires small amounts of DNA, and much less time to be performed (the Fast, SYBR-assay format takes under one hour). This method appears to be sensitive enough even for identification of single *NEO *insertions. Of the two assays tested, the MGB-assay is the least convenient, as it requires two specific and expensive fluorescently-labelled probes, and does not show any practical advantage over the SYBR-assay.

Approaches based on real-time PCR to validate ES positive clones have been previously described, using absolute quantification of NEO, or a relative quantification to detect deletion of the target gene [9,10]. Our method, based on a relative PCR quantification, can be easily reproduced in other laboratories using different technical platforms, and does not need the preparation of standards.

One possible drawback may be the genomic DNA quality/degradation that needs to be checked in case of non-reproducible data.

In conclusion, our real-time PCR assay to quantify *NEO *copy number is a valid alternative tool to Southern blot for the rapid screening of large numbers of ES cell clones during the production of knockout or knockin mouse models.

## Competing interests

The authors declare that they have no competing interests.

## Authors' contributions

CM and EM carried out laboratory experiments, analyzed data and drafted the manuscript; ET analyzed data and revised the manuscript; AB and AB designed the experiments, analyzed data, drafted and revised the manuscript.

All authors read and approved the final manuscript.
